# Barriers and facilitators to colorectal cancer diagnosis in New Zealand: a qualitative study

**DOI:** 10.1186/s12875-020-01276-w

**Published:** 2020-10-01

**Authors:** Tania Blackmore, Kimberley Norman, Jacquie Kidd, Shemana Cassim, Lynne Chepulis, Rawiri Keenan, Melissa Firth, Christopher Jackson, Tim Stokes, David Weller, Jon Emery, Ross Lawrenson

**Affiliations:** 1 Medical Research Centre, University of Waikato, Hamilton, New Zealand; 2 Auckland University of Technology, Auckland, New Zealand; 3 Dunedin School of Medicine, University of Otago, Dunedin, New Zealand; 4 Department of General Practice and Rural Health, University of Otago, Dunedin, New Zealand; 5 Centre for Population Health Studies, The University of Edinburgh, Edinburgh, Scotland UK; 6 Medicine, Dentistry and Health Sciences, The University of Melbourne, Melbourne, Australia

**Keywords:** Colorectal cancer, Delays, Patient-physician relationship

## Abstract

**Background:**

New Zealand (NZ) has high rates of colorectal cancer but low rates of early diagnosis. Due to a lack of understanding of the pre-diagnostic experience from the patient’s perspective, it is necessary to investigate potential patient and health system factors that contribute to longer diagnostic intervals. Previous qualitative studies have discussed delays using The Model of Pathways to Treatment, but this has not been explored in the NZ context. This study aimed to understand the patient experience and perception of their general practitioner (GP) through the diagnostic process in the Waikato region of NZ. In particular, we sought to investigate potential barriers and facilitators that contribute to longer diagnostic intervals.

**Methods:**

Ethical approval for this study was granted by the New Zealand Health and Disability Ethics Committee. Twenty-eight participants, diagnosed with colorectal cancer, were interviewed about their experience. Semi-structured interviews were audio recorded, transcribed verbatim and analysed thematically using The Model of Pathways to Treatment framework (intervals: appraisal, help-seeking, diagnostic).

**Results:**

Participant appraisal of symptoms was a barrier to prompt diagnosis, particularly if symptoms were normalised, intermittent, or isolated in occurrence. Successful self-management techniques also resulted in delayed help-seeking. However if symptoms worsened, disruption to work and daily routines were important facilitators to seeking a GP consultation. Participants positively appraised GPs if they showed good technical competence and were proactive in investigating symptoms. Negative GP appraisals were associated with a lack of physical examinations and misdiagnosis, and left participants feeling dehumanised during the diagnostic process. However high levels of GP interpersonal competence could override poor technical competence, resulting in an overall positive experience, even if the cancer was diagnosed at an advanced stage. Māori participants often appraised symptoms inclusive of their sociocultural environment and considered the impact of their symptoms in relation to family.

**Conclusions:**

The findings of this study highlight the importance of tailored colorectal cancer symptom communication in health campaigns, and indicate the significance of the interpersonal competence aspect of GP-patient interactions. These findings suggest that interpersonal competence be overtly displayed in all GP interactions to ensure a higher likelihood of a positive experience for the patient.

## Background

New Zealand (NZ) has one of the highest rates of colorectal cancer (CRC) in the world. CRC is NZs second most common cause of cancer mortality with over 1200 deaths per annum from around 3000 registered cases [1]. Māori, the indigenous population, are 30% less likely to be diagnosed with CRC but their mortality rates are only slightly lower than NZ European [2]. NZ has a low rate of early stage CRC diagnosis by international standards [3]. Those diagnosed with early stage (I and II) CRC have a better prognosis - at 90% 5-year survival -than those diagnosed with late stage disease (III or IV), at 14% 5-year survival [4]. However, the proportion of Māori and Pacific peoples who have metastatic CRC at diagnosis is much higher than for NZ European (Māori: 31.6%, Pacific: 34.9%, non-Māori/non-Pacific: 22.8%) [5]. These inequities have a considerable and disproportionate impact on poorer outcomes.

Aside from bowel screening, which began gradual regional implementation from 2017 but at the point of this writing has not yet been fully implemented nationwide, improving timely diagnosis is the most important step in ensuring that CRC patients have a better chance at survival [6]. Previous research (the PIPER project) [5] has extensively examined the management of CRC in NZ post-diagnosis and highlighted the need for increased understanding of patient and health system delays prior to diagnosis. Indeed, a NZ Health and Disability Commissioner report (2004–2013) [7], has documented an over-representation of CRC among cancers with longer diagnostic intervals, with the longest times to diagnosis occurring in primary care [7]. Contributing factors to general practitioner (GP) related delay were a lack of clinical examinations and the non-specific presentation of CRC symptoms. Recent research with Māori communities has indicated continuity of care with a trusted GP is needed for general practice to engage better with Māori patients [8].

International studies have indicated that patient, physician and health system delays are key factors associated with late stage diagnosis of CRC. A qualitative study of 20 men in Australia, for example, found delays were associated with patient misinterpretation of symptoms, a failure to attribute symptoms to cancer, and subsequent delays in consulting a health care professional [9]. Other studies have also linked longer diagnostic intervals to CRC symptoms, which are commonly associated with more benign conditions such as irritable bowel syndrome or haemorrhoids, patient-GP communication about symptoms, public and GP awareness of CRC, and hospital system delays in referral and scheduling of colonoscopies [9–11].

Due to the high mortality rates of CRC in NZ and a lack of understanding of the pre-diagnostic experience from the patient’s perspective, it is necessary to investigate the potential barriers and facilitators of CRC diagnosis. Previous qualitative studies have discussed patient and system related delays to diagnosis using The Model of Pathways to Treatment (MPT) [9, 10, 12, 13] but this has not been explored in the NZ context. We report here the qualitative component of a larger study investigating delay and increasing access to early diagnosis for CRC (HRC 17/147). The aim of the current study was to understand the NZ patient experience during the CRC detection period, with a focus on barriers and facilitators to diagnosis.

## Method

### Participants

The 28 participants in this study were previously surveyed as part of a broader quantitative study and had indicated their willingness to take part in an interview. All participants had been diagnosed with CRC within the previous year (study period from 2016 to 2019). They were recruited either through mail out or referral from a CRC cancer nurse specialist at one of the regional district health boards (DHBs) involved in the study (e.g., Waikato, Lakes and Tairawhiti DHBs).

Participants were purposively sampled to obtain representation across key groups (e.g., ethnicity, gender and those who had, and had not, experienced a long interval to diagnosis, as determined by the earlier quantitative study). Three delay intervals were calculated, guided by the Aarhus statement - a guideline for reporting time intervals in cancer-diagnosis research [14], and a previous study [15]. The appraisal/help-seeking interval was determined from patient-reported first symptom recognition (when body changes or symptoms are first noticed) to the date of first presentation to GP or emergency department (ED) admission (when a clinician can start investigations or referral), the diagnostic interval was calculated from the date of first GP consult or ED admission to date of diagnosis (defined as date of first confirmation of cancer) and the total interval was taken as the date of first symptom onset to date of diagnosis. Delay in each of these intervals was defined as > 3 months and no delay was classified as < 3 months, based on a previous review [16]. Participants who were diagnosed through an incidental finding (*n* = 3) or other (usually monitoring (*n* = 1) were not included in delay interval calculations. Participants resided in the midland region of NZ. Ethical approval for this study was granted by the New Zealand Health and Disability Ethics Committee (Ref: 17/NTB/156).

### Data collection

Potential participants were initially contacted via telephone and invited to take part in the qualitative phase of the study. A convenient time and day were arranged to meet for interview. Interviews were usually carried out at the participant’s home and were held from May–December 2019. Written and verbal consent had already been obtained from the earlier quantitative study, but additional verbal consent was also obtained and recorded via audio device immediately prior to commencement of the interview.

Interviews were semi-structured. Before the interview commenced, the objective of the study was restated and study information was read, with an emphasis on the participants’ rights and confidentiality. Māori participants had the option of opening the interview with prayer (karakia), and a culturally driven process of building rapport between the interviewer and participants was followed (whanaungatanga). Participants were thanked for agreeing to participate and compensated with a $30 travel voucher for their time. All interviews were conducted by the same female interviewer (KN) and directed by an interview guide (see supplementary material).

During the interview, participants were invited to speak about their experience of being diagnosed with CRC. A particular focus of the interview was to hear their experiences of symptoms, the timeline from first symptom recognition to diagnosis, their experiences with their GP and their awareness of CRC symptoms prior to diagnosis. All participants were invited to speak about any other information significant to their experience. No time limits were placed on interview duration. Interview data were recorded via audio device, and recordings were transcribed verbatim by the interviewer. All participants were offered the opportunity to review or amend their interview transcripts, however, no participants undertook a review.

### Analytical framework

**Fig. 1:**
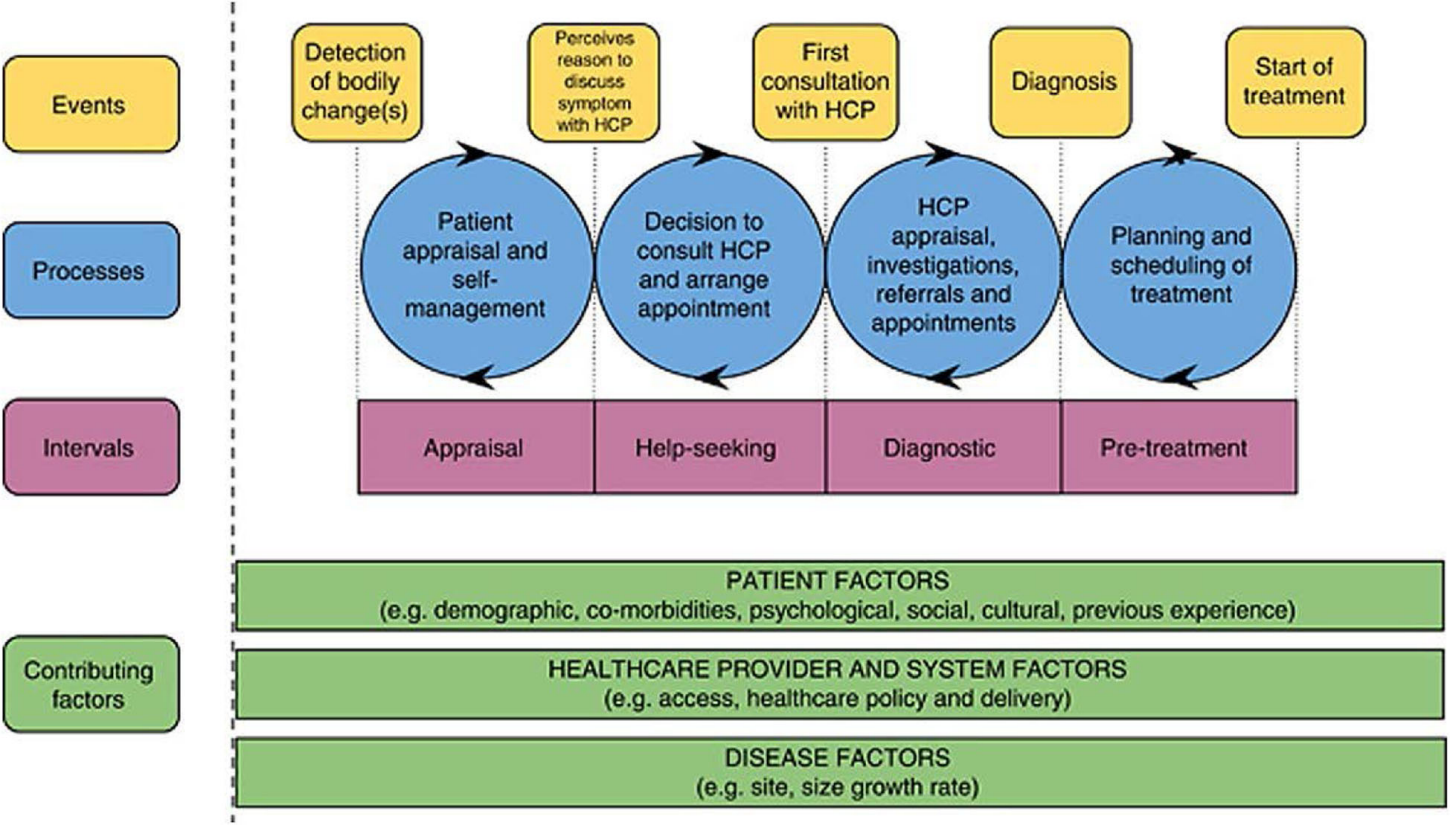
The Model of Pathways to Treatment [17]

The MPT [17] was used as a theoretical framework for the development of the interview schedule and data analysis. The MPT defines four intervals from first symptom/bodily change to commencement of treatment (appraisal, help seeking, diagnostic, and pre-treatment) (see Fig. 1). These intervals are influenced by factors relating to the patient, healthcare provider and system, and disease. This study focused on the first three intervals of the MPT: appraisal, help seeking, and diagnostic. The fourth interval, pre-treatment, was not the primary focus of this study and has been covered elsewhere [5]. Initial coding by the interviewer identified barriers and facilitators to diagnosis. Codes were then grouped into themes based on the MPT model. The Māori data were analysed collaboratively between the interviewer (KN), a qualitative research colleague (SC) and a Māori researcher (JK). Findings are reported according to COREQ guidelines for qualitative research (see supplementary material).

#### Findings

Findings are presented as an overall summary of the participants who experienced delay and those who experienced no delay, followed by rich data within each of the MPT phases and their subthemes. In the appraisal interval the subthemes were self-appraisal and self-management, symptoms worsen was a subtheme in the help-seeking interval, the diagnostic interval subthemes were other diagnoses and patient appraisal of GP. Table 1 shows the characteristics of all participants interviewed. At the time of the interviews, the age of participants ranged from 42 to 86. Cancer stage was obtained from clinical records. Nineteen participants were non-Māori and nine were Māori. The most common patient-reported first symptom was bleeding, followed by changes of bowel habit (COBH). Most participants had been diagnosed through investigations arranged by their GP. Almost 60% of all participants experienced a longer total interval, and over half of all Māori patients experienced a longer total interval. Interviews were not extended beyond 28 participants as data saturation had been reached.

**Table 1:** Participant characteristics (*n* = 28)

Characteristic		n	%
**Gender**	Male	15	53.6
	Female	13	46.4
**Age**	< 40	0	0.0
	40–49	4	14.3
	50–59	2	7.1
	60–69	11	39.3
	70–79	7	25.0
	80+	3	10.7
	Unknown	1	3.6
**Ethnicity**	Non- Māori	19	67.9
	Māori	9	32.1
**First symptom**	COBH	5	17.9
	Bleeding	9	32.1
	Pain	4	14.3
	Weight loss	2	7.1
	Anaemia	2	7.1
	Other	3	10.7
	None	3	10.7
**Mode of detection**	Through my GP	17	60.7
	Incidental finding	3	10.7
	Presented to ED	5	17.9
	Other	1	3.6
	Unknown	2	7.1
**Stage**	I	5	17.9
	II	10	35.7
	III	9	32.1
	IV	2	7.1
	Unknown	2	7.1
**Total interval**	No delay	10	35.7
	Delay	16	57.1
	Unknown	2	7.1
**Appraisal/Help-seeking interval**	No delay	16	57.1
	Delay	7	25.0
	Unknown	5	17.9
**Diagnostic interval**	No delay	13	46.4
	Delay	12	42.9
	Unknown	3	10.7

#### Appraisal interval

##### Self-appraisal

The first theme identified was self-appraisal. All symptomatic participants engaged in a period of symptom self-appraisal, which determined whether or not they consulted a GP. Self-appraisal typically began upon first symptom recognition, whereby the severity of that symptom was appraised and perceived either as ‘normal’ (i.e., similar to a previously experienced symptom) or abnormal (i.e., not previously experienced). If symptoms were normalised, participants typically felt unalarmed, and a GP was less likely to be consulted. One participant normalised their tiredness due to a vegetarian diet, and decided that a GP was not warranted:

*But I’ve been vegetarian for about 15 years, and I’ve always had a naturally low blood iron level. (Male, stage 3)*

Others attributed COBH to previous experiences of stomach ulcers or psychological conditions:

*I have always had a funny guts for, you know years, and years and years … before that I’d actually had a stomach ulcer. So I thought, oh probably something like that. (Male, stage 2).*

*I brushed my diarrhoea off to a large extent, because I knew how my stomach reacts to, tension and stress. (Female, stage 4)*

A GP was also not consulted if a symptom was perceived as an isolated case (e.g., just one bout of bleeding) or if participants attributed symptoms to a benign health issue. For example, if symptoms could be explained by factors such as recent dietary change, changes in exercise routine, stress, lack of fitness, diverticulitis, haemorrhoids, stomach ulcers or emotional tension, a GP was often not consulted immediately. One participant attributed food intake as being responsible for the blood in her stool:

*Often, I used to, when I wipe my behind, I often used to look at it and think, mmm- is there a sign of red in that? But then it was persimmons season, and it was summer we‘d been eating a lot of salads. Is it the beetroot, is it the tomatoes, is it the persimmons? I always found another excuse. (Female, stage 4)*

In contrast, when participants perceived their symptoms as abnormal (e.g., excessive bleeding from the bowel), a GP was more likely to be consulted. One participant assessed bleeding as a stark contrast to their usual bowel habits, which facilitated immediate help-seeking:

*It was just blood, everywhere, and the water just turned bright red … So I went up to the hospital. The emergency department. (Male, stage 4)*

Many of the Māori participants included the impact of their symptoms on their sociocultural environment in their self-appraisal. In particular, symptoms were perceived as less concerning if they could stay private, but once the symptoms became obvious to others around them, they decided to seek advice.

*I kind of put my head down on my desk and my work colleague he walked past and he says, hey you! You better get to the doctors. You look terrible he says. You look like crap! I said thanks for that! (Female, stage 3)*

*Sometimes when I was at work, I couldn’t make it [to the toilet] and um, you sort of um, dirty underwear sort of thing. So changed my underwear every, twice a day, as it got really embarrassing you know? You are too frightened to sit down and have a smoko with the rest of the mates. And you know, they whether they could smell you, I don’t know, but- (Male, stage 3)*

For all the participants, symptoms such as abdominal pain, unexplained weight loss and nausea were perceived as abnormal, and so facilitated a faster GP consultation than other symptoms.

##### Self-management

Self-management was a second theme identified in the appraisal interval. Once symptoms had been appraised, participants employed various self-management techniques. Self-management was usually informed by the type of symptom experienced, the participant’s perception of their own level of health literacy and their previous experience of self-managing symptoms. Self-management ranged from over the counter medication (e.g., for symptoms such as diarrhoea, constipation, and nausea), to dietary or exercise routine changes, to simply waiting for psychological stress to abate:

*I have some diarrhoea tablets to stop the diarrhoea. (Male, stage 2)*

*It was bad diarrhoea. But, um, with the excitement of booking all our holiday and everything I just thought ‘oh its excitement, it will disappear once all that’s done’. (Female, stage 4).*

Self-management and self-appraisal were closely related behaviours. While self-managing, self-appraisal was commonly revisited as participants monitored the progress of the self-management strategies they were employing. Self-management, if successful, resulted in delayed help-seeking if participants felt symptoms had subsided to a more manageable level and therefore did not require professional medical help.

#### Help- seeking interval

##### Symptoms worsen

During the help-seeking interval, the worsening of symptoms was an example of how severe symptoms had to get before a GP was consulted, so was an important facilitator to help-seeking. Self-management was often a temporary strategy, as participants not only reported the return of symptoms, but also usually experienced a pronounced increase in severity whereby symptoms became hard to manage (e.g., if medications were no longer being effective, or dietary changes no longer relieved bowel habits or pain):

*My symptoms weren’t improving in fact I think … just made it worse, you know, so I noticed a lot more. (Male, stage 3)*

For some participants, it was an increase in the number of additional symptoms that warranted cause for concern and facilitated a GP consultation. One participant reported beginning with manageable symptoms that did not cause alarm, such as loss of appetite, however, as time progressed, additional symptoms presented and became unmanageable, prompting a GP consultation:

*In November, a year previously, I, um started having, weight loss and loss of appetite. [Then a while later] either constipation or diarrhoea [so I] went to my local doctor. (Female, stage 4)*

For another participant, the smell associated with bloody stools prompted him to see his GP:

*The smell is the one that probably sticks out the most because it, it just, just lingers aye. It just sits on your tongue like ‘ugggh’. (Male, stage 4)*

Some participants also recognised that symptoms had become unmanageable in their daily routine, as indicated by a change in their physical ability to perform usual household tasks, jobs or manage holidays. One participant reported a lack of energy for any non-work areas of life and another participant outlined the disruption a lack of control over bowel movements caused to a working holiday:

*My life consisted of going to work and coming home and getting my nightie on and going straight to bed. Every night. (Female, stage 4)*

*While I was over there, the pressures like going to the toilet, um was, chronic, and sometimes I’d go, and, and I’d go back to class and then, you know, 15 minutes later I think ‘Oh god I gotta go again!’ (Male, stage 3)*

One participant reported that he was managing his symptoms initially, however once symptoms worsened, he was unable to complete his work efficiently and had to be close to a toilet throughout the day:

I was going to the toilet around about 10 times a day then, and then um, it got worse. I was going 30 / 40 times a day … It was a nuisance. Like, I’d be up on the bloody roof [working, and think] Oh sh**! Down the ladder, into the portaloo – you know? (Male, stage 2)

In this interval the Māori participants were more likely to consider the impact of their symptoms in relation to their families. This included overcoming their concerns about needing to accept help:

*You know in the mirror and you’re like that’s me, because I want to feel positive aye and I want to have pride aye. You know. I have a two year old daughter that um, man I want her to look up to me like, yeah ‘churr my dad’ she would like that. (Male, stage 3)*

*I don’t want to wait until later and write down, and go through all those emotions. Um, when I am meant to be strong for my children … I want to be there for that. (Male, stage 4)*

Disruption to work and inability to manage a daily routine were important facilitators to seeking help for both Māori and non-Māori participants, and was an indicator that self-management options were exhausted/no longer effective and that their health was in a more serious state than initially thought.

#### Diagnostic interval

##### Other diagnoses

A prominent theme identified in the diagnostic interval was the participants’ perception that their symptoms had been misdiagnosed, either once or multiple times. Common misdiagnoses included haemorrhoids, menopause, diverticulitis, vitamin B12 deficiency, low iron, diabetes, stress, anxiety, irritable bowel syndrome, kidney stones and food poisoning, with GPs typically prescribing medication for these.

*Symptoms probably were, around about 10 months prior, um, to finally being diagnosed, and I’d been to my GP quite a few times of that 10 months period with my concerns, and his first comment was, you know ‘it’s probably just piles, you’ve probably just got piles.’ And I said ‘look, I’ve had them before, I know what pile bleeding is’ … I said, ‘This is quite a lot of blood’. (Female, stage 3)*

*I went back to the doctor and I said I’m a little bit concerned you know I’ve got this weight loss and I can’t understand it. I’m still eating. Although I don’t have a great appetite. But um, I’m noticing there’s blood in my stools. And he said to me ‘oh, do you think you might have piles?’ (Female, stage 4)*

*He [doctor] just thought I had irritable bowel syndrome and gave me medication for that which actually made me sick. (Female, stage 3)*

Other diagnoses were reported more often by participants who experienced linger diagnostic intervals (excluding those who were diagnosed incidentally) and therefore was an important barrier to prompt diagnosis.

##### Patient appraisal of GP

Participants typically appraised their GPs performance throughout the diagnostic interval. If they perceived a high level of technical competence (i.e., medical knowledge, performing a physical examination, being proactive, following up on referrals) a positive diagnostic experience was reported, but if participants perceived a poor level of technical competence, then they were more likely to report a negative diagnostic experience.

Participants universally reported a positive experience if their GP investigated symptoms proactively, leading to a prompt diagnosis. For example, some participants praised GPs for having a high level of CRC knowledge (recognising symptoms) and taking the initiative in providing healthcare (referring for colonoscopies / blood tests and calling participants for routine check-ups). One person perceived a high level of technical competence from their GP:

*I did go to my GP. And um, she did some blood tests and I was extra low in iron. So she gave me some iron. Um which made me feel a whole lot better. But in, in between times, she had already written to have a colonoscopy for me to have at [hospital]. Yeah so it’s, she obviously suspected something wasn’t quite right, you know, for losing all that iron out of my body so, yeah. So she then, got things cracking and she really did. (Female, stage 3)*

While the perception of a technically competent GP was associated with prompt diagnosis, a perceived lack of technical competence was an important barrier to diagnosis. For example, a lack of technical competence was perceived if GPs failed to perform appropriate medical examinations before offering a diagnosis. Several participants reported a lack of scans or rectal examinations:

*And I was sent home because she said I had constipation … no scan, no nothing. (Female, stage 3)*

*He seemed to think I had piles, although he didn’t check. He never once, he never once examined me at all. Which I thought was really odd. (Female, stage 4)*

*But, I- in some ways, I think my doctor did fail, yeah, by lack of checking...he could have checked for haemorrhoids. (Female, stage 4)*

In addition to the perception of technical competence, participants also assessed their GPs level of interpersonal competence based on their experiences of feeling respected, informed and cared about. Participants who reported having an overall positive diagnostic experience also perceived their GP to have a high level of interpersonal competence. Interestingly, interpersonal competence could often override perceptions about technical competence and a longer interval to diagnosis, and could still lead to a positive diagnostic experience:

And in the interim again [waiting for non-urgent colonoscopy] we tried to- still tried to identify triggers and we tried to get another anti-nausea thing, that type of thing. Yeah so the on-going care, was, was happening, but not effective … So then, J*** who’s my GP, said okay well let’s try some, we will do some more blood tests etc and this time they did, an iron test … I’ve got the same GP I’ve been seeing for years, yeah, very, very good. (Male, stage unknown)

*He [doctor] said ‘you are under my care’. And that made a big difference, because it showed that somebody actually did care. I wasn’t just a number. (Female, stage 4)*

In contrast, a failure to demonstrate interpersonal competence generated a negative diagnostic experience:

*He just didn’t really care, wasn’t interested and just, look-looked me up and down and just kept typing on his, on the computer. (Female, stage 3)*

For one person, despite having received five earlier non-cancer diagnoses, experiencing a longer interval to diagnosis and cancer progression, it was the perceived lack of interpersonal competence that had the most negative impact:

*I stood at the reception and I, was actually treated quite disrespectfully, through this whole journey. Even by the receptionist because I think, I think they thought I was a hypochondriac … [So I said tell the doctor] I won’t be in for my B12 shot next week because I, I’m, I don’t have B12 deficiency. I have cancer. And I’ve never heard from them. Not an apology. Not a letter. Nope, nothing … and I just feel sorry for anybody else that’s been treated by him because we were just. We were just, I, you know I, I really feel that. Um, that particular company, just, get you in and out. Here’s some drugs, bugger off. We really don’t care. You know? And so all through this, I actually started seeing, I went and got counselling. (Female, stage 4)*

While many of the participants described GP delays as frustrating or worrying, their more emotional descriptions of poor care tended to include incidences where they felt dismissed, ignored or disrespected.

## Discussion

This study sheds light on the barriers and facilitators experienced by CRC patients who either did or did not experience a longer interval to diagnosis. For all the non-Māori symptomatic participants, the perception of an abnormal or previously unexperienced CRC symptom acted as a key facilitator to help-seeking behaviours. However, there was a barrier for some Māori participants who appraised their symptoms according to whether they were perceptible to their work colleagues or family. For all participants, self-managing and normalising symptoms acted as a barrier as no alarm was experienced. Symptoms worsening and an increasing inability to perform routine daily activities was identified as a key facilitator for the majority of symptomatic participants. This was particularly the case for Māori participants, who focused on their desire to involve their children as they made the decision to seek medical help. Other diagnoses being offered before clinical investigations, and a patient-appraised lack of GP technical competence acted as barriers to a prompt CRC diagnosis, whilst in contrast, a perceived high level of technical competence was found to be a facilitator to diagnosis. The perception of interpersonal competence was found to be a key facilitator to diagnosis and dictated the overall positive or negative GP-patient experience.

The symptoms experienced by participants align with the current international literature [18] however, participants in this study reported that the worsening of symptoms had an additional psychosocial effect (inability to socialise, perform employment tasks, or holiday adequately) which acted as a facilitator to consulting a GP. This additional effect is not one that is defined nor measured during a GP consultation, it was found to be a significant facilitator to CRC diagnosis. Further, Māori participants clearly identified the sociocultural context as central to their decision making about whether symptoms were severe enough to warrant medical investigation. This represents an important opportunity for improving cultural safety communication in primary health care if GPs recognise help-seeking behaviour as an indicator of significant patient distress. Further investigation into communication discrepancies is necessary to ensure any potential delays are reduced in this stage of the CRC diagnostic process by developing an understanding of what drives people to seek medical help.

Failure to examine the patient was found to be a significant barrier to CRC diagnosis, and generated a negative overall experience. Participants perceived the absence of physical examinations (commonly for haemorrhoids) as a demonstration of a lack of technical competence in their GPs. This was further evidenced by the participant receiving a diagnosis of ‘piles’ along with prescribed medication, both of which contributed to a longer diagnostic interval. A combination of GP professional processes of diagnosing (differential diagnosis) with the way in which symptoms of CRC are commonly found to be present with other benign bowel diseases could offer a potential explanation as to why non-cancer diagnoses were offered. The appraisal of GP competence is a complex finding, nonetheless, further investigation, and improved access to diagnostic procedures such as colonoscopy for GPs is needed, especially considering the suggestion that NZ GPs have generally more limited access compared to other countries [19].

Interpersonal competence was significant in all patient narratives and dictated whether participants had a positive or negative diagnostic experience. Interestingly, a GP displaying high levels of interpersonal competence could override poor technical competence in producing an overall positive diagnostic experience, even when the cancer was advanced. This finding indicates that interpersonal competence is more important to the patient than technical competence during the diagnostic process. However, the GP-patient relationship was significantly weakened if the GP was appraised as being technically incompetent in addition to not communicating that they cared about the patient.

### Comparison to other literature

This study supports previous literature which indicates that barriers to CRC diagnosis are influenced by the nature of CRC symptoms and the individualised symptom experience [9, 18, 20] along with health literacy levels [21]. However, this study opposes the perspective that patients misinterpret their symptoms [9] which leads to a longer diagnostic interval. Instead, this study offers evidence that patients misattribute, not misinterpret, their CRC symptoms. The definition and measurement of CRC symptoms in some cases do not align. Clear communication in GP-patient consultations is significant and supports previous literature that unclear communication could be an influencing factor between early and late stage diagnosis [10]. In the cases where the symptom definition between patient and GP aligned, the attribution was towards CRC by GPs and non-CRC by patients.

Facilitators to GP consultation and CRC diagnosis identified in this study also support previous literature. Normalising of symptoms by participants acted as a barrier and delayed help-seeking [22]. Symptoms becoming alarming (bleeding from bowel [23]), symptoms becoming unmanageable and a routine disruption were all reported by patients to be key facilitators to GP consultation [12, 15, 18]. This study also offers the perspective of Māori participants, indicating the central position of the sociocultural environment during the symptom appraisal and help-seeking intervals. Clear differences in how indigenous peoples view symptoms and cancer care has also been shown for Aboriginal people in Australia [24]. As found in other NZ studies [25], the GP was the most common point of contact for patients seeking help for CRC symptoms and is seen as crucial for clear CRC information and communication [26, 27]. This competence appraisal strengthened or weakened the GP-patient relationship, which offers support for previous literature that demonstrates trust and positive GP-patient relationships are key facilitators in CRC diagnosis and treatment [10, 27, 28].

### Implications

Overall, the findings from this research hold broader implications relating to the health promotion, health campaign, and CRC symptom education contexts in NZ. Tailoring CRC health messages and information to the non-clinical and culturally diverse audience is crucial for CRC symptoms to be recognised and diagnosed quicker, as recommended by previous literature [23, 29]. This study recommends that CRC health campaigns that ask if one has anaemia will not have any contextual meaning to a non-clinical individual. Instead, this research suggests asking if one is too tired to carry out their normal daily activities, or if their routine has changed due to bowel habits, as this could be a more effective way of generating CRC symptom awareness in individuals and communities with no clinical terminology knowledge. This ‘culturally diverse’ messaging should have a particular focus on Māori and Pacific groups to eliminate inequities in CRC outcomes. A further strategy to emerge from this study is to heighten GPs understanding of the complex appraisal and psychological processes patients go through before seeking a consultation to avoid colluding with incorrect interpretation of symptoms (e.g., the normalising of symptoms). Building awareness across the community would also contribute to GPs being consulted quicker. Having a medical workforce that is more appreciative of the effort it takes many patients to seek help will also make them more likely to listen to what may appear as vague symptoms. These together will enable CRC diagnosis to occur at earlier stages and likely reduce CRC deaths in NZ.

In addition, a key message is the importance of interpersonal and technical competence. Minimising the perception of a lack of technical or interpersonal competence could strengthen GP-patient relationships. Consequently, this could reduce the amount of reported complaints to the Health Commissioner about GPs failure to examine or adequately perform GP duties in the future.

### Strengths/future directions

A major strength of this study was that the patient was enabled the space to speak about their diagnostic experience from their perspective, with Māori participants able to contribute their stories in a culturally safe manner. Whilst this is a qualitative study and findings cannot be generalised, the findings support the broader quantitative research project by providing a more comprehensive understanding of the appraisal, help-seeking and diagnostic intervals that lead to CRC diagnosis. Another strength was the range of participants included in this project, including age, gender, cancer stage and geographical location across the Waikato. Future directions could include a more focussed investigation into (1) the differences in CRC symptom discourse between clinical and non-clinical perspectives and (2) the experiences and processes employed for self-treatment by individuals, as this research identified these two contexts to be significant in the CRC diagnosis experience.

## Conclusion

The findings of this study help to understand the lived experience of the CRC diagnosis in the NZ population as well as identify barriers and facilitators present in the diagnostic experience. These findings indicate a significance of tailored CRC symptom communication in any future health campaigns, as well as indicating the significance of the interpersonal competence aspect of GP-patient interaction, which can generate a positive diagnostic experience despite delays in diagnosis and repeated misdiagnosis. These findings suggest that interpersonal competence be overtly displayed in all GP interactions to ensure a higher likelihood of a positive GP experience for the patient.

## Supplementary information

**Additional file 1.** Supplementary material. COREQ Checklist. Completed COREQ checklist.

**Additional file 2.** Supplementary material. Interview guide. Interview guide used.

## Data Availability

The data analysed for the current study are not publically available for ethical reasons. Anonymised data can be made available from the corresponding author on request.

## Abbreviations

NZ: New Zealand
CRC: Colorectal cancer
GP: General practitioner
MPT: The Model of Pathways to Treatment
DHB: District health board
ED: Emergency department
COBH: Changes of bowel habit
