# Supplementary material for: Barriers and facilitators to colorectal cancer diagnosis in New Zealand: a qualitative study
Source: BMC Fam Pract. 2020 Oct 1;21:206. doi: 10.1186/s12875-020-01276-w (PMC7530960; doi:10.1186/s12875-020-01276-w)
Supplement: Supplementary file 1 — Additional file 1. Supplementary material. COREQ Checklist. Completed COREQ checklist. [file 12875_2020_1276_MOESM1_ESM.docx]

Consolidated criteria for reporting qualitative studies (COREQ): 32-item checklist

Domain 1: Research team and reflexivity

Personal Characteristics

1. Interviewer/facilitator. Which author/s conducted the interview or focus group?

*RESPONSE: one interviewer conducted all interviews. This has been clearly stated in the* ***Data collection*** *section of the* ***Method****.*

2. Credentials. What were the researcher's credentials? E.g. PhD, MD

*RESPONSE: we did not feel it was relevant to report on the interviewer’s credentials. She has a masters degree, all other researchers involved have a PhD or higher.*

3. Occupation. What was their occupation at the time of the study?

*RESPONSE: we did not feel it was relevant to report on the interviewer’s occupation. She was a research assistant at time of the study.*

4. Gender. Was the researcher male or female?

*RESPONSE: we have now stated that the interviewer was female in the* ***Data collection*** *section of the Method.*

5. Experience and training. What experience or training did the researcher have?

*RESPONSE: the interviewer had previous experience from her master’s research, which involved both qualitative interviewing and data transcription. We did not feel it was relevant to report this.*

Relationship with participants

6. Relationship established. Was a relationship established prior to study commencement?

*RESPONSE: we have stated in the* ***Data collection*** *section of the* ***Method****: Before the interview commenced, the objective of the study was restated and study information was read, with an emphasis on the participants’ rights and confidentiality. Māori participants had the option of opening the interview with prayer (karakia), and a culturally driven process of building rapport between the interviewer and participants was followed (whanaungatanga).*

7. Participant knowledge of the interviewer. What did the participants know about the researcher? e.g. personal goals, reasons for doing the research

*RESPONSE: all participants had previously participated in a quantitative interview, so were aware of reasons for doing the research and had met a researcher.*

8. Interviewer characteristics. What characteristics were reported about the interviewer/facilitator? e.g. Bias, assumptions, reasons and interests in the research topic

*RESPONSE: at the start of each interview, the researcher introduced herself and reminded the participant of why we were doing the research. The researcher shared her interests during the interview if it was appropriate.*

Domain 2: study design

Theoretical framework

9. Methodological orientation and Theory. What methodological orientation was stated to underpin the study? e.g. grounded theory, discourse analysis, ethnography, phenomenology, content analysis

*RESPONSE: the Model of Pathways to Treatment was used as a theoretical framework for the development of the interview schedule and data analysis. Thematic analysis was used to analyse transcribed interviews. This is stated in the* ***Analytical Framework*** *section of the* ***Method****.*

Participant selection

10. Sampling. How were participants selected? e.g. purposive, convenience, consecutive, snowball

*RESPONSE: We have stated in the following in the* ***Method: Participants*** *section: Participants were purposively sampled to obtain representation across key groups (e.g., ethnicity, gender and those who had, and had not, experienced a long interval to diagnosis, as determined by the earlier quantitative study).*

11. Method of approach. How were participants approached? e.g. face-to-face, telephone, mail, email

*RESPONSE: We have stated in the following in the* ***Method: Participants*** *section: The 28 participants in this study were previously surveyed as part of a broader quantitative study and had indicated their willingness to take part in an interview. All participants had been diagnosed with CRC within the previous year (study period from 2016-2019). They were recruited either through mail out or referral from a CRC cancer nurse specialist at one of the regional district health boards (DHBs) involved in the study (e.g., Waikato, Lakes and Tairawhiti DHBs). In the* ***Data collection*** *section: Potential participants were initially contacted via telephone and invited to take part in the qualitative phase of the study. A convenient time and day were arranged to meet for interview. Interviews were usually carried out at the participant’s home and were held from May-December 2019.*

12. Sample size. How many participants were in the study?

*RESPONSE: this has been clearly stated in the* ***Participants*** *section of the Method.*

13. Non-participation. How many people refused to participate or dropped out? Reasons?

*RESPONSE: no participants refused to participate or dropped out at the point of being approached for qualitative interview. Hence we do not report this.*

Setting

14. Setting of data collection. Where was the data collected? e.g. home, clinic, workplace

*RESPONSE: this has been clearly stated in the* ***Data collection*** *section of the Method.*

15. Presence of non-participants. Was anyone else present besides the participants and researchers?

*RESPONSE: no.*

16. Description of sample. What are the important characteristics of the sample? e.g. demographic data, date

*RESPONSE: this has been clearly stated in the* ***Findings*** *section of the Results and in* ***Table 1****.*

Data collection

17. Interview guide. Were questions, prompts, guides provided by the authors? Was it pilot tested?

*RESPONSE: an interview guide was used to guide the interview. We have amended the* ***Data collection*** *section of the* ***Method*** *to reflect this.*

18. Repeat interviews. Were repeat interviews carried out? If yes, how many?

*RESPONSE: no, no repeat interviews were necessary.*

19. Audio/visual recording. Did the research use audio or visual recording to collect the data?

*RESPONSE: yes. This has been clearly stated in the* ***Data collection*** *section of the* ***Method****.*

20. Field notes. Were field notes made during and/or after the interview or focus group?

*RESPONSE: as the interviews were audio recorded and later transcribed, field notes were not necessary.*

21. Duration. What was the duration of the interviews or focus group?

*RESPONSE: the duration of interviews varied, and could go up to 2 hours.* *Participants were invited to speak freely, without time limit. We have stated this in the* ***Data collection*** *section of the* ***Method****.*

22. Data saturation. Was data saturation discussed?

*RESPONSE: 28 interviews were conducted. Data saturation was reached at that point.*

23. Transcripts returned. Were transcripts returned to participants for comment and/or correction?

*RESPONSE: yes. This has been clearly stated in* *the* ***Data collection*** *section of the* ***Method***

Domain 3: analysis and findings

Data analysis

24. Number of data coders. How many data coders coded the data?

*RESPONSE: there were 3 data coders. This has been stated in the* ***Analytical Framework*** *section of the* ***Method.***

25. Description of the coding tree. Did authors provide a description of the coding tree?

*RESPONSE: no. there were many codes extracted from the data, and not all were necessarily relevant. Only directly relevant themed codes were used.*

26. Derivation of themes. Were themes identified in advance or derived from the data?

*RESPONSE: we had some idea of themes to expect as a result of the previous quantitative work carried out with the same participants. However, additional themes were derived after analysis of the transcribed data.*

27. Software. What software, if applicable, was used to manage the data?

*RESPONSE: not applicable*.

28. Participant checking. Did participants provide feedback on the findings?

*RESPONSE: as part of participant consent, participants had the choice to indicate whether they would like to receive feedback on the findings at study end. Participants who indicated they would like feedback will be provided with a summary of findings at the end of the study.*

Reporting

29. Quotations presented. Were participant quotations presented to illustrate the themes / findings? Was each quotation identified? e.g. participant number

*RESPONSE: yes. These are clearly shown in the* ***Results*** *section.*

30. Data and findings consistent. Was there consistency between the data presented and the findings?

*RESPONSE: yes. This has been discussed.*

31. Clarity of major themes. Were major themes clearly presented in the findings?

*RESPONSE: yes. These have been clearly outlined in the* ***Results*** *section.*

32. Clarity of minor themes. Is there a description of diverse cases or discussion of minor themes?

*RESPONSE: all relevant themes, major or minor, have been included.*
