# Supplementary material for: Barriers and facilitators to colorectal cancer diagnosis in New Zealand: a qualitative study
Source: BMC Fam Pract. 2020 Oct 1;21:206. doi: 10.1186/s12875-020-01276-w (PMC7530960; doi:10.1186/s12875-020-01276-w)
Supplement: Supplementary file 2 — Additional file 2. Supplementary material. Interview guide. Interview guide used. [file 12875_2020_1276_MOESM2_ESM.docx]

**Interview Guide**

**Introduction – Whanaungatanga**

- Thank the participant for their time and participation
- Ask about Karakia
- Introduce self/background including where from etc
- Respond to participants own introduction
- Answer and questions and ask participant if it’s okay to start the interview

**Recap of Research Aims**

- Explain aims of the research and interview
- Verbally go through the information sheet and consent form, answer any questions/ clarify and concerns and give participant a copy of the information sheet and consent form
- Gain audio recorded consent for turning Dictaphone on

**Interview Questions**

1. Could you please tell me about your experience of being diagnosed with colorectal cancer?

- When? Who diagnosed this (GP / Family / Hospital)? Knowledge of colorectal cancer prior to diagnosis? Knowledge of the cancer pathway? What were you told about at key phases?

1. Could you please tell me about what happened in the time leading up to your diagnosis, so from the time you recognised something wasn’t right to when you sought healthcare or advice from your GP?

- Nature of the symptoms (what were they)? First symptom recognition (what was it that first alerted you something wasn’t right)? Timing between the symptom and seeking health care

1. Could you please tell me about your experience of when you had contacted your GP or Healthcare professional after you had recognised you were unwell?

- What was the health seeking symptom/s? Relationship with GP – or other health care professionals? What support was given by your health care professional / GP? Describe your experiences of healthcare leading up to the diagnosis? Healthcare engagement (any barriers / enablers from your perspective)?

1. Can you please tell me about how you found out more information about your condition?

- What were they health seeking actions/ behaviours? People or services contacted for advice? Supportive care (referral and knowledge)? Self- management?

**End of Interview**

- Ask the participant if there is anything they would like to add to their narrative / experience
- Thank the participant for their time and participation
- Ask the participant if they would like to do a closing karakia
